# A Novel Third Wave Contextual Approach of Positive Behavior Support in School for Adolescent at High Psychosocial Risk: Rationale, Feasibility, and First Pilot Outcomes

**DOI:** 10.3389/fpsyg.2019.02635

**Published:** 2019-11-29

**Authors:** Flavia Marino, Ilaria Crimi, Cristina Carrozza, Chiara Failla, Stefania Trusso Sfrazzetto, Paola Chilà, Marilla Bianco, Antonino A. Arnao, Gennaro Tartarisco, Angelo Cavallaro, Liliana Ruta, David Vagni, Giovanni Pioggia

**Affiliations:** ^1^Institute for Biomedical Research and Innovation (IRIB), National Research Council of Italy (CNR), Messina, Italy; ^2^Italian National Institute of Health, Rome, Italy; ^3^Istituto Comprensivo “G. Catalfamo”, Secondary School, Messina, Italy

**Keywords:** adolescents, psychosocial risk, prevention, intervention, borderline intellectual functioning, DNA-V

## Abstract

Adolescence is a stage in life when dramatic physical, cognitive and socio-emotional changes occur. When adolescents grow-up in deprived social environments, the chance of psychophysical well-being severely decreases and problems such as delinquency, substance abuse and mental health issues are much more likely to ensue. Third wave cognitive-behavioral interventions are increasingly becoming the chosen instruments to support psychological intervention for young people and adolescents. In this study, we aim to test the feasibility and the adequacy of the outcome measures of an intervention for adolescents at high psychosocial risk, using a modified Discoverer, Noticer, Advisor and Values (DNA-V) protocol aimed at increasing flexible and positive values. The project was conducted in a school located in a low Socio-Economic Status (SES) and severely deprived district of a metropolitan area in Messina, Italy, with 3 classes from 6th to 8th grade. All parents and teachers allowed participants to take part in the pilot study. However, the participants’ willingness to engage in the study was low (1 out of 3 classes). Overall, 13 adolescents (72% of the enrolled class) participated in the pilot and only 2 out of 7 teachers and no parents were available for interviews. In its current form, a full RCT is not considered feasible due to general low motivation showed by the participants. Although the sample size was small, the intervention program showed a statistically significant main effect for students’ self-report questionnaire, suggesting that those measures were appropriate. Modifications and additional measures are suggested to increase participants’ engagement and to overcome the need for parents and teachers’ interviews.

## Introduction

Globally (Polanczyk et al., 2015), approximately one in eight children or adolescents experience a mental disorder. The prevalence increases during adolescence and young adulthood. Additionally, the Global Burden of Disease Study 2015 (GBD 2015 Disease and Injury Incidence and Prevalence Collaborators, 2016) estimated that one third of the top 25 causes of years lived with disability (YLD) globally were mental disorders. Many intervention studies target high-risk individuals or children of wealthy and educated families who are eager to participate in research studies. Nevertheless, mental health issues are spread throughout the population and contact with educational and prevention services can easily be increased through school-based interventions.

In an effort to translate laboratory research into the actual social context, we started a collaboration with a school located in a peripheral area of Messina, Italy, in which the socio-economic status (SES) is particularly low. According to the Italian Ministry of Education^1^, there is a substantial deficit between the standardized test results for students of class 2 of the upper secondary schools in Messina, Italy, compared to the national average. In addition, there are many other factors that worsen the general socio-economic situation of the city. One such example is the disposable income per capita in families, which is lower than the national average as well as the heterogeneous distribution of low and high-income neighborhoods.

The larger research project focused on bullying and the rate of school drop-outs within a culturally and economically impoverished social context. It is worth mentioning that within the school environment, respect and sharing of ideas among the students, cooperation, and prosocial behaviors were low. Furthermore, informal and anecdotal evidence showed that social stigma of mental health issues is still high while access to mental health services is low. Nowadays, public health interventions often use a resilience-based approach for school interventions. A recent meta-analysis of social-emotional interventions by Taylor et al. (2017) suggested that significantly better results can be reached within experimental groups in terms of social-emotional skills, attitudes, and indicators of well-being in comparison with control groups when using resilience-based interventions within the framework of positive psychology. Positive psychology is the scientific field of study aimed at “identifying strengths and skills that enable individuals and communities to thrive” (Brockman et al., 2017). Further, positive psychology is considered a complex and heterogeneous field that aims to enhance skills such as resilience, engagement, positive emotions and social connectedness (Chodkiewicz and Boyle, 2017).

In the meta-analysis, post interventions aimed at the development of social-emotional skills. They showed significant results in terms of prediction of well-being and improvement of critical aspects of students’ developmental trajectories at follow-up. Results demonstrated independence from students’ race, socioeconomic background, and school location (Taylor et al., 2017).

Resilience, the ability to recover from and adjust to unfortunate events or unexpected changes, allows an individual to become stronger and healthier, and is also a fundamental skill to have during the transitional period of adolescence (Dray et al., 2017).

Nevertheless, when considering resilience-based interventions, it is recommended to pay attention to the socio-economic environment of the subjects, as well as the individual features of the population of interest (Fergus and Zimmerman, 2005). For example, in hostile and unsupportive social environments (e.g., schools, neighborhoods), adolescents at high psychosocial risk should be supported in the process of building resilience in order to prevent mental illness and to develop higher cognitive, social, and emotional skills that can help them connect meaningfully with their communities. Thus, in order to design a mental health prevention strategy in adolescents at high psychosocial risk, a training program should be designed to enhance: (1) resilience, (2) cognitive skills, (3) social skills, and (4) emotional skills. However, adolescents that were born and live in hostile and unsupportive social environments may never have experience a positive social reference to follow, and these adolescents can show several problems in adaptive functioning, especially in social situations and in peer relations (Ciarrochi et al., 2016). That is why, noticeably, when adolescents grow-up in a severely deprived social environment, the chance of a double-hit increases, with neurodevelopmental disorders and mental health problems immediately following social stressors. In fact, one of the worst possible consequences of living in a deprived condition is the long-term development of aggressive and antisocial behaviors, often paired with psychiatric disorders, delinquency, and substance misuse in adulthood (Giovanelli et al., 2016). Aggressive and antisocial behaviors represent major mental health issues during youth (Van Nieuwenhuijzen and Vriens, 2012; Schuiringa et al., 2017). Also, they are often related to hostile attribution bias and aggressive responses.

### Choosing the Right Intervention

In order for a resilience-based intervention to work within social context of a low socio-economic social context, it is pivotal to provide adolescents with the cognitive and emotional tools needed to cope with their environment. Growing-up in a deprived environment usually comes with a lower emotional knowledge, a reduced social support, and a lack of personal values and aims linked to a sense of helplessness (Esposito, 2003). In such an environment, in order to heal, it is important to foster not only resilience, but also positive values, acceptance, and a worthy sense of self. The recommended approach to enhance those skills is Acceptance and Commitment Therapy (ACT) (A-tjak et al., 2015).

People usually develop ideas about themselves throughout their lives. However, this process tends to be particularly noticeable during childhood. ‘Stories’ people have about themselves are necessary as they help maintain some sense of self-coherence. However, these self-descriptions can limit these children and reduce their ability to grow if they consider the stories to be true, negative, and unchangeable. One term to describe this aspect of the self is the self-as-a-content or “conceptualized self” (McHugh and Stewart, 2012). Another aspect of the self is the idea of experiencing what is happening in each moment. This is the active subset of people that can feel and take action. This aspect is called self-as-a-process or “experiential self” (McHugh and Stewart, 2012). The third self is the Observer or “self-as-a-context” (McHugh and Stewart, 2012). This last part of the self is the context in which all of these thoughts and feelings occur. This part of the self is the one that is aware that people are able to notice their own thoughts, as well as the one that has been consistent all through their life, even though they have grown and changed. People who have coped resiliently with traumatic events often talk about connecting with this part of themselves: “I realized that there is a part of me that cannot be hurt by painful thoughts, feelings and memories or even outside circumstance” (Collins, 2011).

There is a substantial lack of literature regarding the use of ACT with adolescents. However, a few studies have been conducted that implemented full ACT protocols with teenagers, suggesting the possibility for an adaptation. Several studies showed that experimental methods based on mindfulness-based therapy with adolescents can have positive effects on reducing anxiety, somatic distress, and depressive symptoms, as well as increasing self-esteem and sleep quality (Biegel et al., 2009; Ames et al., 2014). Mindfulness-based treatments were administered to adolescents affected by Autism Spectrum Disorders (ASD) in order to lower the frequency of aggressive behaviors (Singh et al., 2011) and also administered to youths diagnosed with Attention Deficit/Hyperactivity Disorder (ADHD) in order to improve attention and cognitive inhibition (Zylowska et al., 2008). Similarly, a treatment method mimicking the defusion strategy of an ACT protocol was experimented with using adolescents at behavioral risk as subjects. The selected youths were involved in defusion exercises such as awareness of breathing, neutral and problematic thoughts, among others (Luciano et al., 2011). Results of the study by Luciano et al. (2011) can be summarized in a lower number of problematic behaviors and in the improvement of psychological flexibility and acceptance without judgment.

Thus, there is evidence suggesting benefits in applying third generation social interventions stemming from ACT as contextual positive psychology. Furthermore, ACT can be effectively adapted and applied with adolescents with high psychosocial risk in a school setting.

In order to bring positive psychology to young students at school, it is necessary to focus on their relationship with their social environment (the external context). In this case, positive personality traits like positive affect, optimism and tenacity (grit) are no longer considered as intrinsic to the person, but as internal content of thoughts and feelings depending on one’s personal background. Therefore, the ability to observe the content of their thoughts and choose their own reaction to them can be seen as an internal context in which those positive traits can be fostered. That is why a context-focused positive intervention might help overcome some of the main criticisms of positive psychology, such as the fact that the individual circumstances seem to be ignored too often. This intervention would be overcome by bringing together the external and internal context to foster personal change and value-based action on the social domain. Context-focused positive interventions are aimed at assisting adolescents in effectively responding to their environment with flexibility rather than aggression, reaching their full potential with actions that are personal, meaningful and important.

There are several protocols with the aim of interweaving ACT principles with the regular school system (Gillard et al., 2018). In particular, the DNA-V protocol (Discoverer, Noticer, Advisor and Values) (Hayes and Ciarrochi, 2015) is a collection of methods and techniques dedicated to support teachers, counselors, and educational professionals in promoting the psychological well-being of typically developing adolescents. Moreover, considering that the DNA-V model is based on an adaptation of the ACT Hexaflex model, it is suitable for working both with children and young people. In fact, DNA-V was repeatedly tested in cohort studies with teenagers outside the school context (Hayes et al., 2011; Livheim et al., 2015). Such papers support the evidence that DNA-V improves well-being and psychological flexibility in adolescents. However, the efficacy of this protocol has yet to be tested within the school context.

### Aims and Hypothesis

We hypothesized that the DNA-V group intervention for adolescents may be adapted for students at high psychosocial risk in a school setting. The DNA-V model would enable participants to increase their psychological flexibility, to choose actions that are driven by their own values and to identify and develop their internal character strengths (Hayes and Ciarrochi, 2015).

Nevertheless, possible contextual barriers can reduce the feasibility of a full Randomized Control Trial (RCT). Therefore, with this study, we aimed at answering the following questions:

1. Is the intervention acceptable for teachers, parents and students?
2. Will the students participate actively in the developed intervention protocol?
3. Will the stakeholders participate actively in the intervention process and gathering of outcome measures?
4. Are the outcome measures and self-reports used in the literature for DNA-V and mindfulness intervention adequate for our specific context?

We assessed whether or not it would be feasible to conduct an RCT of the DNA-V-based intervention in schools for adolescents at high psychosocial risk. The focus was on: (1) the recruitment and capability of the students to properly complete the DNA-V-based intervention at school; (2) adequacy of outcomes measures; and (3) evaluating the acceptability of intervention, adherence, participant retention, and treatment fidelity. Feasibility and pilot study were reported as the preliminary planning of a forthcoming full-size RCT.

## Materials and Methods

### Participants

For the feasibility study, three classes (6th, 7th, and 8th grade) were enrolled. The full pilot protocol was administered to 13 adolescents (nine females and four males) with a mean age of 13 (age range 12–14 y.o.) who were enrolled in the study. All participants completed the assessment. The group was homogeneous in term of intellectual functioning, and all were in the 8th grade. All the participants were affected by borderline intellectual functioning or mild intellectual disability with a median Intelligence Quotient (IQ) of 73 and an Interquartile Range (IR) of 72–74 (Table 1).

**TABLE 1:** Sample characteristic and outcome measures.

**Demographics**				**Outcome measures**			
	
**Id**	**SPM-IQ**	**Age in years**	**Gender**	**AFQ-Y Pre**	**AFQ-Y Post**	**CAMM Pre**	**CAMM Post**
1	70	12	F	26	33	19	20
3	71	14	F	23	26	25	21
4	72	13	F	29	25	28	21^∗^
5	75	14	F	33	20	25	28
7	74	14	F	30	17	19	17
9	75	12	F	31	23	27	19
10	72	13	F	32	31	12	14^∗^
11	72	13	M	19	16	33	19
14	73	14	M	25	12	^∗∗^	^∗∗^
15	73	12	F	26	22	32	31
16	71	12	M	30	31	19	24^∗^
17	73	14	M	29	25	25	19
18	76	13	F	39	20	14	22
Mdn	73 (72–74)	13 (12–14)	9F:4M	29 (26–31)	23 (20–26)	25 (19–27)	21 (19–23)
Mean	72.8 (1.7)	13.1 (0.8)	–	28.6 (4.8)	23.2 (6.0)	23.2 (6.3)	21.3 (4.4)

*Mdn, median, in parentheses the interquartile range or the standard deviation; SPM-IQ, intelligent quotient with Raven’s standard progressive matrices; AFQ-Y, acceptance and fusion questionnaire for youth; CAMM, child and adolescent mindfulness measure; ^∗^ one missing item. Score prorated multiplying it by 10/9 and rounded to the nearest digit; ^∗∗^ six missing items in the pre-test CAMM questionnaire, participant excluded.*

### Ethics Statement

The study was conducted in accordance with the relevant guidelines and regulations for human subjects. The study design was approved by the Research Ethics and Bioethics Committee^2^ of the CNR. All participants’ parents signed a written consent form. Participants were recruited as part of a larger and ongoing research program about school drop-outs tested by CNR of Messina.

### DNA-V Based Group Intervention Protocol Adapted for Adolescents at High Psychosocial Risk

The original DNA-V program is a psychoeducational group intervention for adolescents delivered by trained psychologists and divided into six sessions (Hayes and Ciarrochi, 2015; Rayner et al., 2017). In this study, the protocol was delivered by clinical psychologists and a CBT psychotherapist. Teachers were not involved in any part of the process of the administration of the DNA-V.

The DNA-V model is used to provide concrete contextual interventions through methods and instruments dedicated to train practitioners to manage the active elements of an intervention protocol for adolescents, such as mindfulness-based and cognitive behavioral approaches and social and emotional learning programs. The first three words represent different functional classes of behavior within a context comprising both the self and social environments. DNA classes of behavior are used to build values: the V in the acronym. In particular, the Noticer is a functional class of attending behavior strictly connected with our own inner experience and related to the physical stimuli coming from the external environment. The Noticer helps us consider the external physical signals as simply messages. Avoiding a subject may push them as well as cling to them. The Advisor metaphorically represents our own “inner voice,” which provides us with a support for judgments, beliefs, evaluations, and sense of ourselves and others. It allows a safe adaptation to the world, without the need for trial and error learning. Conversely, the Discoverer function is aimed to allow the world to be engaged by a subject through trial and error learning, making it useful for understanding how to interact and functionally adapt to it. DNA-V is a positive psychology intervention model dedicated to train the practitioner to develop young people’s skills in the use of D, N, and A classes of behavior, improving their flexibility, adaptability, competences in shift behaviors and in functioning on the basis of their values, constraints and demands. DNA-V is aimed at helping subjects to reach their own full potential in a way that is consistent with their values and flexible in the process of adapting to their living conditions.

The first session is an introduction to the program, while the following sessions are each dedicated to a certain function (notice, adviser, discover) of the protocol. Subsequently, each session is dedicated to a set of values. The final session is dedicated to self-view, well-being, and closure.

In a set of preliminary meetings, the protocol was agreed to with the school head teacher and regular teachers, then communicated to parents and other school personnel, in order to obtain the necessary authorizations and ethical consents. Two other meetings aimed at gathering qualitative feedback from the class teachers were held after the sixth session, and 1 week after the end of the protocol (for qualitative data, see section “Feasibility and Qualitative Data”).

As already has been mentioned, the protocol was presented within the context of a bullying reduction and prevention program to increase the acceptability for the school system. This is because it was expected that an increase in self-acceptance, resilience, and mindful attention could decrease aggressive behaviors (Singh et al., 2007). The original DNA-V protocol was extended in order to account for the specificity of the population studied.

In the first and preliminary session, the protocol objectives were presented to the class and some simple activities were planned to increase student trust and bonding with the psychologists. The second session was based on standard Cognitive Behavioral Therapy (CBT) procedures of emotional literacy, focusing on basic emotional recognition, emotional labeling, and context-to-emotion association (e.g., “How do I feel when…?”). The third session was a brief CBT assertiveness training focused on the recognition of the four-communication styles (assertive, passive, aggressive and passive-aggressive) and the specific posture, non-verbal signal, voice prosody and words that identify them. From the fourth session to the end, the content of the original DNA-V protocol was used.

A decision was made to start the protocol with the sessions related to values, instead of administering them at the end. This was because goal-directed behavior during negative emotions is considered the most reliable predictor of mental health and well-being during adolescence (Forsyth, 2016). Furthermore, goals and values can differ between individuals (Schwartz et al., 2012; Hayes et al., 2013; Mennin and Fresco, 2014; Hayes and Ciarrochi, 2015). As a result, working with young people at the beginning to identify their goals and values should feasibly help them learn about themselves, and connect with the personal meaning of any regulatory skills training that is subsequently introduced. Values allow individuals maintain contact with what is important to them. Values can be identified by the questions: “What is important for me?,” and “What is meaningful?”. Those questions were operationalized through the use of Value Cards as reported by Juarascio et al. (2013).

The sixth and seventh sessions focused on the Adviser that allowed us to identify the participants’ inner thoughts. The Adviser was introduced through the use of visual metaphors, creative writing exercises, and extremization/normalization procedures to foster defusion (Masuda et al., 2006).

The eighth and ninth sessions introduced the Observer. Usually, formal and informal mindfulness exercises are used. However, this study focused mainly on informal exercises (e.g., the “mindful eating of a candy”) deemed more acceptable by the students as they considered formal meditation a “silly practice for privileged people.” In this context, mindfulness is used to foster the link between interoceptive messages from the body, the outside environment, and emotions.

The tenth and eleventh sessions focused on the Discoverer, in order to find alternative behaviors and new reinforcement in the context in which they are living. For those sessions, metaphors, worksheets (“My travel through values”) and Personal Strengths Cards (Hayes and Ciarrochi, 2015) were employed.

The last session was kept as the original protocol: self-view, well-being and closure. A sum-up of the different sessions is outlined in Table 2.

**TABLE 2:** Research timeline and activities.

**Feasibility study**		**Pilot study**	
	
***Weeks***	**Activities**	***Weeks***	**Activities**
*–*	Meeting and planning with school head	–	–
*1*	School wide presentation of the project	–	–
*2–4*	Parents and teachers consent, willingness to participate	–	–
*5*	Class observation, students consent and willingness to participate, teachers’ pre-intervention interviews	*1*	Protocol objectives and some simple activities presented to the class
*6*	Observation during assessment	–	Students IQ and pre-intervention assessment
*7*		*2*	Emotional literacy and emotions recognition
*8*		*3*	Assertive training focused on the recognition of the four-communication style
*9–10*		*4–5*	DNA-V: identify personal goals and values
*10–11*	Management and feedback meetings with teachers and school head	*5–6*	DNA-V: inner thoughts, creative writing, and procedures to foster defusion
*12–13*		*7–8*	DNA-V: informal mindfulness exercises
*14–15*		*10–11*	DNA-V: find alternative behaviors and new reinforcement, personal strengths
*16*		*12*	DNA-V: self-view, wellbeing and closure
*17*	Teachers post-intervention interviews	–	Students Post-intervention assessment
*18*	Management and feedback meetings with teachers and school head	–	–

### Feasibility and Qualitative Data

The participants’ access level to the intervention was determined in terms of the responses of participants to communication and the proportion of adolescents in the school who met the eligibility criteria. Feasibility was initially studied through qualitative interviews and observations during the protocol’s first session.

In order to gather information on the self-perception of each member of the school class and to assess the feasibility of the outcome measures, both teachers and students who participated in the study underwent semi-structured interviews with the aim of gathering qualitative data (the list of questions asked during the interview can be found in Supplementary Materials).

The interviews were comprised of ten questions aimed at investigating the quality of the classroom context, especially concerning the relationships between students. Those interviews allowed the researchers to analyze: (1) the quality of the social network of the classroom; (2) the existing emotional relationships; (3) students’ collaboration in order to achieve common goals, mutual appreciation and the rules and norms dictating the group’s social functioning. We planned a thematic analysis for the semi-structured interview.

### Pilot Outcome Measures

The pilot outcome measures for all of the participants were completed just before the second session and after the last session of the DNA-V intervention (e.g., baseline evaluation was administered 1 week before the first session; final evaluation was administered 1 week after the last session). They were the: Child and Adolescent Mindfulness Measure (CAMM) (Kuby et al., 2015) and Avoidance and Fusion Questionnaire for Youth (AFQ-Y) (Greco et al., 2008). Both are self-reported questionnaires and were completed under the experimenters’ supervision.

The CAMM is a 10-item measure of mindfulness designed for use with children and adolescents aged 10 to 17, as validated in an Italian sample (Saggino et al., 2017). It conceptualizes mindfulness as a trait more than a state of mind, hypothesizing that in general, individuals tend to mindful at different degrees. CAMM’s aim is to assess aspects of mindfulness such as acting with awareness (e.g., a person’s ability to experience the present moment without evaluating it) (Baer et al., 2008). Items of the CAMM are reverse scored, with lower scores indicating more self-reported mindfulness. This measure has shown good internal consistency (α = 0.80) (Greco et al., 2011), as well as appropriate convergent and divergent validity in an initial validation study (Lechtenberg, 2012).

AFQ-Y was developed as a measure of experiential avoidance in children and adolescents. It is a child report measure designed to assess psychological inflexibility, characterized by high levels of cognitive fusion and experiential avoidance, validated in Italian (Ristallo et al., 2016). Items of the AFQ-Y were intentionally created to use less ACT-specific language in order to be more accessible for adolescents. This self-report scale has 17 items that assess experiential avoidance and cognitive fusion. The AFQ-Y showed adequate internal consistency reliability (α = 0.90) in a validation study (Greco et al., 2008), and in a study with adolescents at high psychosocial risk (Murrell and Kapadia, 2011).

CAMM and AFQ-Y have the same structure. Items are on a Likert-like scale that ranges from 0 (“not at all true”) to 4 (“very true”). High scores indicate low mindfulness and high psychological inflexibility, respectively.

### Pilot Statistical Analyses

All statistical analyses were run with SPSS software (v. 23, IBM Corporation, Armonk, NY, United States).

For the pilot study, considering the small sample size, a non-parametric statistic (Related Sample Wilcoxon signed-rank test) was applied. This allowed us to analyze the effects of groups and intervention(s). We used a one-sided test, because our hypotheses were directional. Single-item responses were used as secondary measures. More than one statistical inference were considered simultaneously. Thus, in order to counteract the problem of multiple comparisons, the alpha-level was adjusted using Šidák correction with the aim of controlling familywise error rate (Šidák, 1967). α = 0.025 for the primary measures: AFQ-Y and CAMM total scores. For the single item analyses: α = 0.003 for AFQ-Y; α = 0.005 for CAMM.

During the initial assessment, we observed a tendency of many participants to respond impulsively. This behavior seemed to increase in frequency as the assessment progressed. As mentioned above, the two questionnaires have the same structure with the lowest score on the left-side of the sheet and no inverse-rated questions. As a result, we hypothesized that with the decrease of sustained attention, the default response style would have been to give the first available answer producing a linear decrease of the items score with time. Therefore, we first assessed the extreme response styles (ERS) (Greenleaf, 1992), comparing extreme responses (0 or 4) with non-extreme responses (1, 2, or 3) for each participant, computing a response style ratio (RSR) as the number of extreme responses divided by the total number of cumulative responses for the two questionnaires (27). We set as lower and upper bound 0.185 and 0.666, respectively; corresponding to a significant (*p* < 0.05) difference for a Chi-squared 2 × 2 contingency table comparing the participant extreme response ratio to the expected (0.400) from a random sample. The Durbin-Watson test was used to test the null hypothesis of zero autocorrelation in the residuals of subsequent items against the alternative hypothesis that the residuals are positively (negatively) autocorrelated at the 1% level of significance (Ostrom, 1990).

Furthermore, we conducted an auxiliary simple linear regression (see paragraph 3.5) aimed at verifying any change in the response style within time. Linear regression was calculated to predict outcome values based on the ordinal item position as a proxy for time (Malhotra, 2008).

## Results

### Feasibility: Process Assessment

**FIGURE 1:**
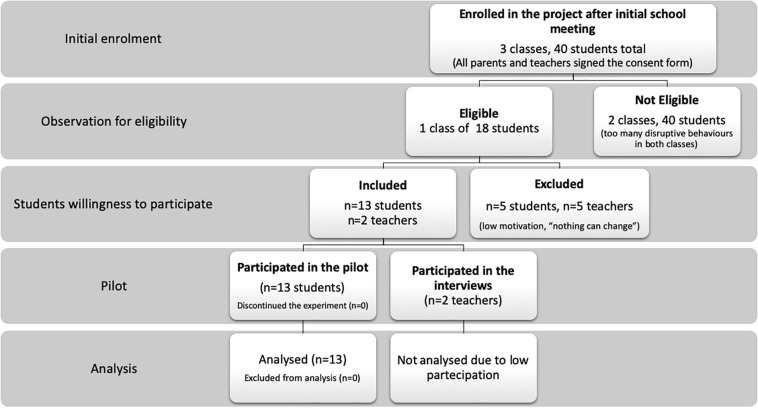
Subjects recruitment, assignment, and assessment procedures.

Finding eligible members of the target population was not difficult, as links with local services can easily point to deprived socio-environmental clusters within any metropolitan region. Recruitment through school personnel ensured starting within the new school year with all of the participants needed. All parents and teachers signed the consent form to allow the participants to take part in the study. At the same time, in terms of the willingness of students to be enrolled in the proposed treatment group, one out of three classes participated. The other two classes had, on average, a level of behavioral problems judged too high by the clinicians’ observations during the first session. From the class that participated in the full pilot, a 72% participation rate (13 out of 18) was obtained. A flow chart of the feasibility process is outlined in Figure 1.

### Management and Scientific Assessment

After consultations with the head teacher and regular teachers, we agreed to use two chartered clinical psychologists and a CBT psychotherapist to administer the protocol. We considered a 1:5 ratio between operators and students as a minimum to (1) administer the protocol, (2) keep track of the progress, and (3) manage the class behavior.

The final protocol consisted of 12 90-min sessions (3 + 9), once per week. Given the population’s characteristics and setting, assessment procedures were selected to ensure a short administration time. Therefore, we selected a short cognitive assessment (Raven’s standard progressive matrices (SPM), with the new Italian standardization (Picone et al., 2016), and two short self-report questionnaires to measure aspects of acceptance and mindfulness (AFQ-Y and CAMM) previously discussed.

The assessment and the outcome measure procedures were both feasible and sustainable. The assessment was finalized within 1 h, with minimal effort and resources.

The therapists showed high adherence to the protocol, as confirmed by blind observers. In terms of participants’ adherence to the intervention, all participating students were present for at least 83% of the sessions (maximum 2 absences). No risk of intervention contamination was observed.

### Demographic Assessment

Assessment and pre-intervention outcome measures were taken before the second session. For the outcome questionnaires, the questions were read aloud by the CBT psychotherapist and every student answered on his own sheet. Participants were allowed to ask clarifying questions at any time during the assessment.

Demographic and clinical characteristics of the participants are reported in Table 1. Pre-intervention outcome measures were slightly higher than normative values for AFQ-Y, Mdn = 29 (26–31), Mdn(*Z*) = 0.599 (Greco et al., 2008), and at the normative value for CAMM, Mdn = 25 (19–27), Mdn(*Z*) = 0.022 (Ristallo et al., 2016). Full responses for each participant are reported in Supplementary Tables S1, S2.

All of the students included in the study had borderline intellectual functioning according to Raven’s Standard Progressive Matrices, Mdn (IQR) = 72 (72–74).

### Qualitative Data Analysis

The teacher’s will to participate in the study was, unfortunately, considerably lower than expected. Only 2 out of 7 class teachers participated. Therefore, it was not possible to complete an exhaustive thematic analysis of the qualitative data.

### Pilot Outcome Measures Assessment

**FIGURE 2:**
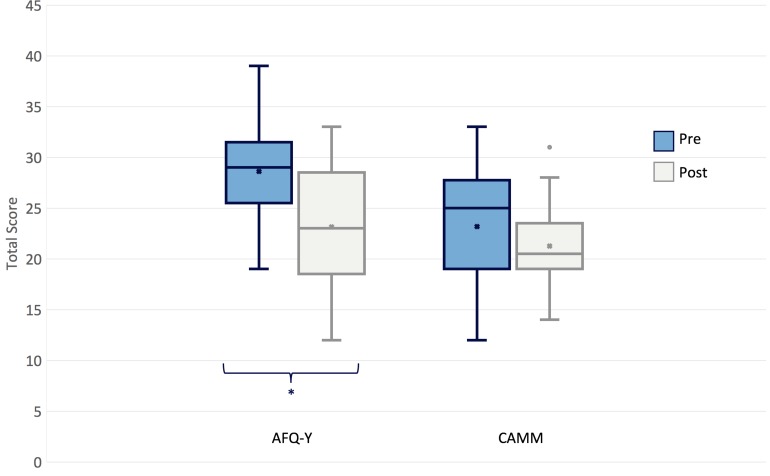
Change in outcome measures pre- and post- intervention.

As shown in Figure 2, the participants displayed a statistically significant improvement on AFQ-Y, *W* = 13.0, *Z* = −2.28, *p* = 0.010, with a mean decrease of 19% in the total score. Conversely, although decreasing by 8%, the CAMM total score did not show any significant change, *W* = 27.5, *Z* = −0.903, *p* = 0.196 (Table 3). Results using a within-subject repeated measure ANOVA showed a similar effect (Supplementary Table S6a).

**TABLE 3:** Change in outcome measures Pre- and Post- intervention.

**Test**		**Difference**			**Related Samples Wilcoxon Signed Rank Test**				
		
**Test**	***N***	**25th**	**50th (Mdn)**	**75th**	***W***	***Z***	***p* (2-tailed)**	***p* (1-tailed)**	**Point probability**
AFQ-Y	13	−13.0	−4.00	−1.00	13.0	−2.28^a^	0.021	0.010	0.001
CAMM	12	−7.50	1.00	4.25	27.5	−0.903^a^	0.392	0.196	0.012

*AFQ-Y, acceptance and fusion questionnaire for youth; CAMM, child and adolescent mindfulness measure; N, number of adolescents; Mdn, median; ^*a*^Based on positive ranks.*

As shown in Table 3, the total score on AFQ-Y for the majority of participants decreased, while more variability was present on CAMM total score.

Nevertheless, at the single item level of analysis, after correction, we found a decreasing, but non-significant, trend for the third item of AFQ-Y, *Z* = −2.33, *p* = 0.011 (Supplementary Table S3), and a statistically significant reduction in the second item of CAMM, *Z* = −2.97, *p* < 0.001 (Supplementary Table S4). Single item analysis is reported in Supplementary Materials.

### Adequacy of Outcome Measures

Every participant completed all of the items of AFQ-Y, while for CAMM, in the pre-intervention condition, one participant did not respond to 6 out of 10 items, was therefore excluded from the subsequent analyses of that measure. 3 other participants did not respond to 1 item each in the post-intervention condition. The total CAMM score was pro-rated using the median value.

For two participants, RSR was below the lower bond in the pre-intervention condition. For one participant, it was below in the post-intervention. The average RSR was 0.315 (0.099) before and 0.320 (0.093) after the intervention. The RSR difference from the expected value for a random distribution was statistically significant both before, *t*(11) = 2.97, *p* = 0.013, and after, *t*(11) = 2.98, *p* = 0.013; while difference in RSR between pre- and post-intervention, *t*(22) = 0.128, *p* = 0.900, was not significant.

**FIGURE 3:**
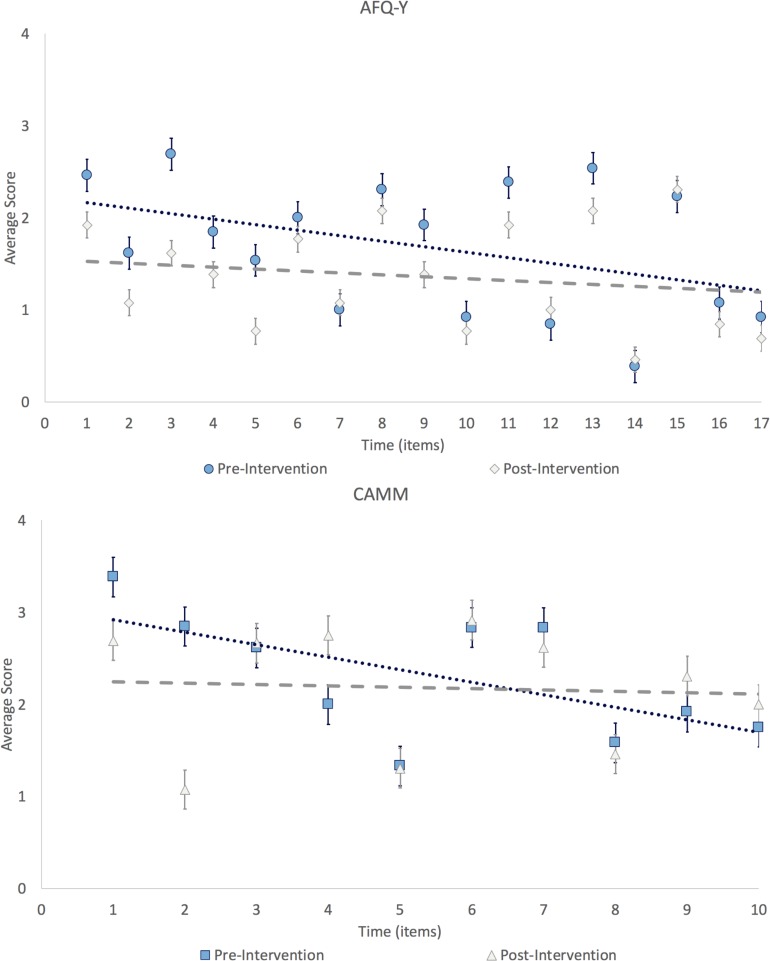
Temporal bias on item responses.

Based on that added linear decrease with time hypotheses, regression equations were found for the tests taken pre-intervention (Figure 3). At AFQ-Y, *F*(1, 219) = 11.8, *p* = 0.001 with an *R*^2^ of 0.047; participants’ predicted response (SD) is equal to 2.16 (0.174) for the first item and the value decrease by 0.059 for each subsequent item. At CAMM, *F*(1, 115) = 12.2, *p* = 0.001 with an *R*^2^ of 0.088; participants’ predicted response is equal to 5.38 (0.916) and the value decrease by 0.141 for each subsequent item. Post-intervention, the regression equations were not significant. At AFQ-Y, *F*(1, 219) = 1.58, *p* = 0.221 with an *R*^2^ of 0.003; participants’ predicted response is equal to 1.53 (0.159) for the first item and the value decrease by 0.021 for each subsequent item. At CAMM, *F*(1, 115) = 0.019, *p* = 0.891 with an *R*^2^ of −0.009; participants’ predicted response is equal to 2.26 (0.913) and the value increase by 0.006 for each subsequent item (Supplementary Tables S5a,b).

*Post hoc* comparisons, of pre and post-intervention measures, using the predicted values from the regression model for AFQ-Y, *t*(24) = 2.84, *p* = 0.009, *d* = 1.11; and CAMM, *t*(22) = 2.49, *p* = 0.021, *d* = 1.01, were statistically significant for both questionnaires (Supplementary Table S5b).

Linear contrasts using a within-subject repeated measures ANOVA shown similar effects (Supplementary Table S6b).

Finally, using Durbin-Watson test, we found no significant autocorrelation between subsequent items in the pre- and post-intervention measures (Supplementary Table S5b).

### Power Needed for a Full RCT

A statistical power analysis was performed for sample size estimation, based on data from the pilot, to compute the number of participants needed for a full RCT. The effect size (ES) for AFQ-Y in the pilot was *d* = 0.994, and ES for CAMM was *d* = 0.350, before adjustment for the linear decrease, considered to be large and small, respectively, using Cohen’s criteria. With an alpha = 0.05 and power = 0.80, the projected sample sizes needed with those effect sizes (GPower 3.1) are approximately *N* = 13 for AFQ-Y and *N* = 102 for CAMM, for this simplest between group comparison using a one-sided equality. Nevertheless, introducing the correction for the linear decrease the sample size needed for AFQ-Y and CAMM decreased to *N* = 10 and *N* = 11, respectively.

## Discussion

We explored the feasibility of a 12-session, psychosocial school intervention to increase self-acceptance, resilience and mindful attention, as well as decrease bullying/aggressive behavior among adolescents at high psychosocial risk in a deprived metropolitan environment. The intervention was adapted from DNA-V (Hayes and Ciarrochi, 2015) and included skills that can enable the development of emotional literacy, psychological flexibility, and engage in values-based actions and develop internal personality strengths and resilience. Qualitative interviews with school personnel and observations with the children were used to assess feasibility, while a non-randomized pilot was used to assess adequacy of outcome measures.

There was not enough qualitative data to be analyzed. Nevertheless, discourse with the institute head teacher and the teachers revealed that families with higher SES are likely to leave the district or send their children to other schools. Furthermore, drop-out rates are high, and many children do not attend school regularly. Criminality, alcoholism, and psychiatric problems are high among parents.

The specific intervention was agreed to by the Institute head teacher, and the feasibility assessed in a secondary school of the same Institute, with a session (class 6th to 8th) amended in the district of the city, with the lowest SES, and the highest rate of criminality. That session revealed a particularly high concentration of students at psychosocial risk.

### Participation and Data Gathering

As mentioned in the results section, acceptability was high among parents and teachers. We should point out that parents accepted the intervention but were not interested in any form of active participation. Only two of seven teachers, participated actively. It was not possible to gather quantitative data about the willingness of parents and teachers to participate in the assessment as they were unwilling to actively do so. That is why we deemed the completing of parents’ and teachers’ report unfeasible.

After the first presentation, the study was considered feasible, only for the latest class. In the other two, behavioral problems were extreme, and acceptability from students too low (it was possible to assess both, only qualitatively). For those classes, working on the social environment, entrusting behavior, all rules, and increasing motivation were considered necessary before any possible study with the DNA-V protocol.

### Protocol Administration and Cognitive Abilities of the Students

Intervention objectives were explained to students in a simple way. They were told that they would follow a path that would support them in choosing socially appropriate behavior.

As noted in the Results section, participants in the pilot had a cognitive level, according to SPM, in the borderline intellectual functioning range. We are unable to obtain clinical diagnoses of participants. However, a summary was reported to the school. Among participants, three had a support teacher for inclusion, in a normal school, according to Italian laws (not present during the protocol administration) and a diagnosis of mild intellectual disability. The other two had a clinical diagnosis of ADHD (Attention Deficit and Hyperactivity Disorder) and ODD (Oppositive-Defiant Disorder).

During the analysis of our data, we noticed that all participants were showing traits of borderline intellectual functioning. Thus, we decided to explore its impact on the participants when exposed to the DNA-V intervention. Borderline intellectual functioning is a boundary status between intellectual disability and typical development and is considered highly heritable (Reichenberg et al., 2016). It does not stem from a neurodevelopmental syndrome, leading to a heterogeneous functioning profile (Contena and Taddei, 2017). It is characterized by a range of cognitive challenges, with an intelligence quotient (IQ) between 70 and 85 points, and a failure to meet developmental and sociocultural standards for personal independence and social responsibility that affect daily activities (Seltzer et al., 2005). This condition is closely associated with failure of social expectations, such as school dropouts, social isolation, inadequate affective relationships, and risk of developing psychiatric disorders in adolescence (Einfeld et al., 2011). Further, during adulthood, it increases the risk of depression, suicidal thoughts, self-harm, emotional distress, anxiety and antisocial disorders (Chen et al., 2006) fostering future socio-economic disadvantage (Gigi et al., 2014). It is also associated with difficulties in social-cognitive skills and social information processing (Van Nieuwenhuijzen and Vriens, 2012; Baglio et al., 2016) that further reduce the general ability to cope. When a child with borderline intellectual functioning grows up in a deprived environment, frequently feedback interaction worsens the outcome, creating an inter-generational cycle and hopelessness in families living in this situation (Hackman and Farah, 2009; Hatton and Emerson, 2009).

To our knowledge, there is no literature on using ACT with adolescents, at high psychosocial risk, and with borderline intellectual functioning. Also, if interaction between mild cognitive impairments, and deprived social environments is not the focus of the study, when planning for a RCT, co-occurrence is a key factor to consider for any complex and contextual intervention, for such a population, and its mediating effect needs further investigation.

### Adequacy of the Outcome Measures

Although the actual effectiveness of the measurements used in the study could not be established due to the small size of the sample, researchers deemed important to report what has been found regarding the adequacy of the outcome measures. The AFQ-Y total score revealed a statistically significant improvement, with a mean decrease of 19%. Also, for CAMM, the total score revealed an improvement, with a mean decrease of 8%. However, the improvement was not statistically significant.

The final sample size was too small to reach the significance level due to the low participation rate. Future replication studies in a school setting should include a larger initial sample to reach the sample size needed for a full RCT.

Further, in the initial assessment, we observed:

1. An increase in impulsive and habitual responses during tests.
2. A higher than expected frequency of extreme response rate.

Nevertheless, the pre- and post-intervention response distribution were comparable and autocorrelation in the residuals of subsequent items were not statistically significant. Therefore, we suggest that those effects were not caused by random responses but may be explained by a decrease of sustained attention, as well as a subsequent increase in default response style (i.e., first responses available in our sample). This behavior may be detrimental to the validity of an RCT. As this effect is present, both questionnaires of 17 and 10 items each, introducing additional control questions, may not be feasible. To reduce the effect, we suggest randomizing the question for each participant, with the expected result of averaging it when comparing mean values. Further, changing the presentation format, using a tablet to answer and proposing single questions at fixed time intervals, could increase response validity by reducing the reinforcing value of finishing the test earlier. Nevertheless, our results suggest a linear decrease in the items score with time. After considering this reduction, both AFQ-Y and CAMM measures showed a statistically significant improvement. The effect was statistically significant only for measures taken pre-intervention. A possible reason for this, to be addressed in a future experiment, could be that (increasing mindful reflection) the intervention reduced impulsiveness and habitual responses. Therefore, if replicated, the reduction of linear score decrease in time could be considered a behavioral outcome measure in itself.

### Study Power

We do not expect any increment in AFQ-Y or CAMM for students without DNA-V. Therefore, we may assume that the effect size for pre- and post-intervention, and for the comparison between intervention group and control group, would be similar. According to the power analysis, a sample size of *N* = 13, or *N* = 10 after linear correction would be adequate for AFQ-Y, while a sample of *N* = 102 or *N* = 11 after correction, would be adequate for CAMM. Thus, a sample size of *N* = 40 could be more than adequate for the main objective of an RCT. It should also allow for expected attrition and our additional objectives of controlling for possible moderating factors and item response analysis. To reach the sample size needed, this study suggests running a future RCT involving more school districts, and in different cities with comparable social environments.

### Attractiveness and Accessibility

To avoid difficulties with the randomization process and possibly ascertain biases due to the low participation rate, it is suggested we increase the attractiveness of the study in the perception of the students. To do so:

1. The study was completed inside the school during normal class periods in order to increase access level, acceptability, and treatment adherence. Qualitative interviews with students revealed that extra hours after school would have reduced the number of students willing to participate, as it would have been perceived as punishment.
2. We plan to gamify the protocol, through the use of technology, to increase engagement. DNA-V is easily adaptable as a card/role playing video game on a tablet. In a series of qualitative interviews with students after the pilot, this concept was found to be attractive.
3. It is important, given the socio-cultural needs expressed by the participants, to find ways that connect the principles of the DNA-V intervention to the daily reality of the sample, in order to make it more interesting and relatable. Moreover, the protocol should aim to be consistent with the participants’ level of development which seems to be mainly focused on practical activities rather than abstract concepts. That is why it is no coincidence that the highest statistically significant effect was found in the most practical item of the CAMM questionnaire. Interestingly, an extremely large effect size, *Z* = −2.97, was revealed for the second CAMM items: “At school, I walk from class to class without noticing what I’m doing.” That item is the most practical (the others are about thoughts and feelings). And, its reduction may be a signpost for an increase in students’ awareness of their environment and engagement in the school system.
4. Future trials can implement a combination of observation schedules, compiled by a blind observer, and secondary data from school conduct registries (suspensions, amends, etc.), to assess deviant or aggressive behavior to complement self-report and teacher-report questionnaires.
5. The students’ clinical characteristics were not directly assessed, but according to the teachers’ reports they were wide and heterogeneous. While heterogeneity of the sample is usually avoided in clinical studies, testing the protocol’s diversity in an inclusive school for educational purposes actually increases the ecological validity of the research.

## Conclusion

A novel modified DNA-V group protocol was applied to high-risk adolescents within a school setting, in a deprived Italian metropolitan area. While the intervention showed the potential to positively influence social behavior and conduct through mindful thinking and value-based actions, the paucity of social and cognitive resources available to the participants are barriers to the implementation prevented highly active participation. Thus, a future RCT of the modified DNA-V intervention presented in the study is not feasible in its current form. Despite this, considerable and valuable insight has been obtained showing the need for a greater embedding in the social and cultural context. Future studies could focus on exploring a possible increase in psychological flexibility, mindfulness and value-driven actions in order to justify an RCT. Further, they could check for signs of reduction in bullying and antisocial behaviors post-intervention. In addition, they could consider the use of contextual, technological and methodological modifications to increase the engagement of the students and other stakeholders. A careful consideration of the cognitive characteristics of the participants is also needed. In our sample, there was a high frequency of neurodevelopmental and mild cognitive impairments. This implies the need for a simplified presentation method of self-reported measures. Furthermore, observational schedules may be used to directly assess behavioral changes in the absence of teachers’ and parents’ reports. Finally, reframing the intervention as a video game could increase the attractiveness and the engagement rate of possible future participants.

## Ethics Statement

The study was conducted in accordance with the relevant guidelines and regulations for human subjects. The study design was approved by the Research Ethics and Bioethics Committee (http://www.cnr.it/ethics) of the CNR. All participants’ parents signed a written consent form. Participants were recruited as part of a larger and ongoing research program about school drop-outs tested by CNR of Messina.

## Author Contributions

FM, IC, CC, AC, and LR designed the study. FM, IC, CC, SS, CF, and PC interviewed parents, professionals, and adolescents for the feasibility. FM, IC, and CC administered the pilot intervention. SS, CF, and PC collected and coded the data, and reviewed the protocol adherence. AA, DV, and GP designed and realized a web platform for data collection and analysis. AC, LR, MB, AA, DV, and GP analyzed the data and wrote the manuscript. All authors read and approved the final manuscript.

## Conflict of Interest

The authors declare that the research was conducted in the absence of any commercial or financial relationships that could be construed as a potential conflict of interest.

## Footnotes

1 http://www.istat.it

2 http://www.cnr.it/ethics

## Abbreviations

ACT: acceptance and commitment therapy
ADHD: attention deficit/hyperactivity disorder
AFQ-Y: avoidance and fusion questionnaire for youth
ASD: autism spectrum disorders
CAMM: child and adolescent mindfulness measure
CBT: cognitive behavioral therapy
CNR: Italian National Research Council
DNA-V: discoverer, noticer, advisor and values protocol
ES: effect size
IQ: intelligence quotient
ODD: oppositive-defiant disorder
RCT: randomized control trial
RSR: response style ratio.
