# Supplementary material for: A Novel Third Wave Contextual Approach of Positive Behavior Support in School for Adolescent at High Psychosocial Risk: Rationale, Feasibility, and First Pilot Outcomes
Source: Front Psychol. 2019 Nov 29;10:2635. doi: 10.3389/fpsyg.2019.02635 (PMC6896099; doi:10.3389/fpsyg.2019.02635)
Supplement: Supplementary file 1 [file Data_Sheet_1.PDF]

Supplementary Material

Qualitative Questions set

1. In the classroom, are the students' ideas respected?
2. Are there any friendships between the students in the classroom?
3. Are there any moments in which you can share your experiences with the class?
4. Can you work in a group with your classmates?
5. Do you think you and your classmates are able to find agreements to solve problems?
6. Do you help each other?
7. Do you help your classmates if they are in trouble?
8. Are you happy with the atmosphere in your class?
9. Do you think you are a good classmate?
10. Are you able to work together towards a common goal?

Supplementary Data

Table S1. Acceptance and Fusion Questionnaire for Youth (AFQ-Y)

	Pre Intervention																	Post Intervention																
**Id**	**1**	**2**	**3**	**4**	**5**	**6**	**7**	**8**	**9**	**10**	**11**	**12**	**13**	**14**	**15**	**16**	**17**	**1**	**2**	**3**	**4**	**5**	**6**	**7**	**8**	**9**	**10**	**11**	**12**	**13**	**14**	**15**	**16**	**17**
**1**	2	1	4	2	1	2	1	2	1	0	1	1	4	0	2	1	1	3	1	2	2	2	3	3	3	2	0	1	0	4	0	4	2	1
**3**	2	1	0	1	3	4	0	2	2	1	2	0	1	0	1	3	0	2	3	1	0	1	3	2	3	1	2	4	1	0	0	2	1	0
**4**	4	1	3	1	0	3	0	2	4	1	2	1	3	1	3	0	0	4	1	2	1	1	2	1	3	4	0	3	0	1	1	1	0	0
**5**	3	1	2	3	1	3	1	2	3	2	3	0	4	0	4	1	0	0	1	2	1	1	1	1	2	1	2	0	1	3	0	3	1	0
**7**	3	1	4	3	1	1	2	3	3	0	3	1	1	0	3	1	0	3	0	1	3	0	1	0	1	2	0	1	2	1	0	2	0	0
**9**	1	3	2	3	3	1	1	3	1	2	3	0	4	0	3	1	0	0	1	0	1	2	1	1	3	1	0	1	1	4	0	4	3	0
**10**	2	1	4	2	0	1	3	3	3	0	1	2	4	1	3	1	2	2	3	1	4	1	3	2	3	2	1	2	0	2	1	3	1	0
**11**	3	2	1	1	0	1	0	0	1	0	2	4	1	0	2	0	1	1	1	2	0	0	0	1	0	1	1	0	2	3	0	4	0	0
**14**	3	1	2	1	0	2	0	3	0	1	3	0	2	0	3	1	3	2	1	1	1	0	2	0	1	1	0	1	0	0	0	0	0	2
**15**	1	3	4	0	3	1	1	3	1	2	3	0	1	0	3	0	0	1	0	4	1	1	1	0	0	1	2	2	3	4	0	2	0	0
**16**	3	1	4	1	3	1	3	3	2	1	3	0	1	1	1	2	0	3	1	2	1	1	3	2	4	1	1	4	1	2	0	4	1	0
**17**	4	1	1	3	4	2	0	3	0	1	2	0	3	2	0	0	3	3	0	1	0	0	2	0	3	0	0	3	2	1	4	0	2	4
**18**	1	4	4	3	1	4	1	1	4	1	3	2	4	0	1	3	2	1	1	2	3	0	1	1	1	1	1	3	0	2	0	1	0	2

Table S2. Child and Adolescent Mindfulness Measure (CAMM)

	Pre Intervention										Post Intervention									
**Id**	**1**	**2**	**3**	**4**	**5**	**6**	**7**	**8**	**9**	**10**	**1**	**2**	**3**	**4**	**5**	**6**	**7**	**8**	**9**	**10**
**1**	2	2	1	3	2	3	2	2	1	1	2	0	3	3	3	0	3	2	3	1
**3**	4	3	4	1	0	4	3	1	3	2	1	3	2	4	0	4	3	1	1	2
**4**	4	4	2	3	1	3	4	2	2	3	1	0	3	4	1	-	3	3	3	1
**5**	4	2	3	4	1	3	4	0	3	1	4	1	3	3	2	3	3	2	3	4
**7**	4	4	3	1	1	3	1	1	1	0	3	0	3	1	3	2	0	1	1	3
**9**	4	2	4	3	1	3	4	2	1	3	3	1	4	2	1	3	2	0	2	1
**10**	2	1	0	2	1	1	1	1	1	2	3	0	2	-	1	1	2	1	2	1
**11**	4	3	2	2	3	4	4	4	3	4	3	0	1	3	1	3	3	1	2	2
**14**	2	3	3	2	-	-	-	-	-	-	3	2	2	4	2	4	3	2	1	2
**15**	4	4	4	3	2	3	4	2	2	4	3	3	4	3	1	4	3	2	4	4
**16**	4	3	3	2	0	2	3	1	1	0	3	0	-	2	1	4	4	2	4	2
**17**	4	3	4	0	1	2	4	3	4	0	4	1	3	0	1	3	4	0	3	0
**18**	2	3	1	0	3	3	0	0	1	1	2	3	2	4	0	4	1	2	1	3

Table S3. Single Item Pre and Post Intervention Comparison for Acceptance and Fusion Questionnaire for Youth

Descriptive Statistics							Percentiles			Related Samples Wilcoxon Signed Rank Test			
Con.	#	N	Mean	SD	Min	Max	25th	50th (Mdn)	75th	*Z*	*p* (2-tailed)	*p* (1-tailed)	Point Probability
Pre	1	13	2.46	1.05	1.0	4.0	1.5	3.0	3.0	-	-	-	-
Post	*1*	*13*	*1.92*	*1.26*	*0.0*	*4.0*	*1.0*	*2.0*	*3.0*	*-1.73b*	*.156*	*.078*	*.063*
Pre	2	13	1.62	1.04	1.0	4.0	1.0	1.0	2.5	-	-	-	-
Post	*2*	*13*	*1.08*	*.954*	*0.0*	*3.0*	*0.5*	*1.0*	*1.0*	*-1.13b*	*.313*	*.156*	*.012*
Pre	3	13	2.69	1.44	0.0	4.0	1.5	3.0	4.0	-	-	-	-
Post	*3*	*13*	*1.62*	*.961*	*0.0*	*4.0*	*1.0*	*2.0*	*2.0*	*-2.33b*	*.021*	*.011**	*.006*
Pre	4	13	1.85	1.07	0.0	3.0	1.0	2.0	3.0	-	-	-	-
Post	*4*	*13*	*1.38*	*1.26*	*0.0*	*4.0*	*0.5*	*1.0*	*2.5*	*-1.20b*	*.328*	*.164*	*.078*
Pre	5	13	1.54	1.45	0.0	4.0	0.0	1.0	3.0	-	-	-	-
Post	*5*	*13*	*.769*	*.725*	*0.0*	*2.0*	*0.0*	*1.0*	*1.0*	*-1.78b*	*.090*	*.045*	*.020*
Pre	6	13	2.00	1.15	1.0	4.0	1.0	2.0	3.0	-	-	-	-
Post	*6*	*13*	*1.77*	*1.01*	*0.0*	*3.0*	*1.0*	*2.0*	*3.0*	*-.499b*	*.703*	*.352*	*.047*
Pre	7	13	1.00	1.08	0.0	3.0	0.0	1.0	1.5	-	-	-	-
Post	*7*	*13*	*1.08*	*.954*	*0.0*	*3.0*	*0.0*	*1.0*	*2.0*	*-.289c*	*.883*	*.441*	*.117*
Pre	8	13	2.31	.947	0.0	3.0	2.0	3.0	3.0	-	-	-	-
Post	*8*	*13*	*2.08*	*1.32*	*0.0*	*4.0*	*1.0*	*3.0*	*3.0*	*-.690b*	*.484*	*.242*	*.008*
Pre	9	13	1.92	1.38	0.0	4.0	1.0	2.0	3.0	-	-	-	-
Post	*9*	*13*	*1.38*	*.961*	*0.0*	*4.0*	*1.0*	*1.0*	*2.0*	*-1.61b*	*.180*	*.090*	*.063*
Pre	10	13	.923	.760	0.0	2.0	0.0	1.0	1.5	-	-	-	-
Post	*10*	*13*	*.769*	*.832*	*0.0*	*2.0*	*0.0*	*1.0*	*1.5*	*-.632b*	*.766*	*.383*	*.203*
Pre	11	13	2.38	.768	1.0	3.0	2.0	3.0	3.0	-	-	-	-
Post	*11*	*13*	*1.92*	*1.38*	*0.0*	*4.0*	*1.0*	*2.0*	*3.0*	*-1.18b*	*.271*	*.136*	*.017*
Pre	12	13	.846	1.21	0.0	4.0	0.0	0.0	1.5	-	-	-	-
Post	*12*	*13*	*1.00*	*1.00*	*0.0*	*3.0*	*0.0*	*1.0*	*2.0*	*-.201c*	*.868*	*.434*	*.021*
Pre	13	13	2.54	1.39	1.0	4.0	1.0	3.0	4.0	-	-	-	-
Post	*13*	*13*	*2.08*	*1.44*	*0.0*	*4.0*	*1.0*	*2.0*	*3.5*	*-.942b*	*.377*	*.188*	*.018*
Pre	14	13	.385	.650	0.0	2.0	0.0	0.0	1.0	-	-	-	-
Post	*14*	*13*	*.462*	*1.13*	*0.0*	*4.0*	*0.0*	*0.0*	*0.5*	*-.447c*	*1.00*	*.500*	*.250*
Pre	15	13	2.23	1.17	0.0	4.0	1.0	3.0	3.0	-	-	-	-
Post	*15*	*13*	*2.31*	*1.49*	*0.0*	*4.0*	*1.0*	*2.0*	*4.0*	*-.207c*	*.887*	*.443*	*.059*
Pre	16	13	1.08	1.04	0.0	3.0	0.0	1.0	1.5	-	-	-	-
Post	*16*	*13*	*.846*	*.987*	*0.0*	*3.0*	*0.0*	*1.0*	*1.5*	*-.499b*	*.703*	*.352*	*.047*
Pre	17	13	.923	1.19	0.0	3.0	0.0	0.0	2.0	-	-	-	-
Post	*17*	*13*	*.692*	*1.25*	*0.0*	*4.0*	*0.0*	*0.0*	*1.5*	*-1.13b*	*.500*	*.250*	*.188*

# = Item number; N = Number of Adolescents;
SD = Standard Deviation; Mdn = Median; Z = Standardized W Test Score;

b = Based on positive ranks; c = Based on negative ranks; p computed with Exact Methods;

**p<.05 but not Significant with Sidak Correction; alpha set to 0.003 with Sidak Correction for Multiple Comparisons.*

Table S4. Single Item Pre and Post Intervention Comparison for Child and Adolescent Mindfulness Measure

		Descriptive Statistics					Percentiles			Related Samples Wilcoxon Signed Rank Test			
Con.	#	N	Mean	SD	Min	Max	25th	50th (Mdn)	75th	*Z*	*p* (2-tailed)	*p* (1-tailed)	Point Probability
Pre	1	13	3.38	.961	2.0	4.0	2.0	4.0	4.0	-	-	-	-
Post	*1*	*13*	*2.85*	*.899*	*1.0*	*4.0*	*2.0*	*3.0*	*3.5*	*-1.81b*	*.113*	*.057*	*.041*
Pre	2	13	2.62	1.33	0.0	4.0	1.5	3.0	4.0	-	-	-	-
Post	*2*	*13*	*2.00*	*1.22*	*0.0*	*4.0*	*1.0*	*2.0*	*3.0*	*-2.97b*	*.001*	*< .001***	*< .001*
Pre	3	12	1.33	.985	0.0	3.0	1.0	1.0	2.0	-	-	-	-
Post	*3*	*12*	*2.83*	*.835*	*1.0*	*4.0*	*2.3*	*3.0*	*3.0*	*-.289c*	*.883*	*.441*	*.117*
Pre	4	12	2.83	1.47	0.0	4.0	1.3	3.5	4.0	-	-	-	-
Post	*4*	*12*	*1.58*	*1.16*	*0.0*	*4.0*	*1.0*	*1.5*	*2.0*	*-1.55c*	*.188*	*.094*	*.055*
Pre	5	12	1.92	1.08	1.0	4.0	1.0	1.5	3.0	-	-	-	-
Post	*5*	*12*	*1.75*	*1.48*	*0.0*	*4.0*	*0.3*	*1.5*	*3.0*	*-.172b*	*.938*	*.469*	*.063*
Pre	6	13	2.69	.947	1.0	4.0	2.0	3.0	3.0	-	-	-	-
Post	*6*	*13*	*1.08*	*1.26*	*0.0*	*3.0*	*0.0*	*1.0*	*2.5*	*-.175c*	*1.00*	*.500*	*.086*
Pre	7	12	2.67	.888	1.0	4.0	2.0	3.0	3.0	-	-	-	-
Post	*7*	*12*	*2.75*	*1.29*	*0.0*	*4.0*	*2.0*	*3.0*	*4.0*	*-.832b*	*.590*	*.295*	*.158*
Pre	8	13	1.31	.947	0.0	3.0	1.0	1.0	2.0	-	-	-	-
Post	*8*	*12*	*2.92*	*1.31*	*0.0*	*4.0*	*2.3*	*3.0*	*4.0*	*-.513b*	*.656*	*.328*	*.023*
Pre	9	13	2.62	1.12	0.0	4.0	2.0	3.0	3.0	-	-	-	-
Post	*9*	*13*	*1.46*	*.877*	*0.0*	*3.0*	*1.0*	*2.0*	*2.0*	*-1.15c*	*.332*	*.166*	*.059*
Pre	10	13	2.31	1.11	1.0	4.0	1.0	2.0	3.0	-	-	-	-
Post	*10*	*13*	*2.00*	*1.22*	*0.0*	*4.0*	*1.0*	*2.0*	*3.0*	*-.719c*	*.594*	*.297*	*.039*

# = Item number; N = Number of Adolescents;
SD = Standard Deviation; Mdn = Median; Z = Standardized W Test Score;

b = Based on positive ranks; c = Based on negative ranks; p computed with Exact Methods;

***p<.005 Significant with Sidak Correction; alpha set to 0.005 with Sidak Correction for Multiple Comparisons.*

Table S5a. Temporal Bias on Item Responses

				Linear Regression: items coefficient							Linear Regression: constant				
Condition	Test	df Hyp.	df Err.	*r*	R^2	*B*	*bias*	*S.E.*	*95% C.I.*	*p*	*c*	*bias*	*S.E.*	*95% C.I.*	*P*
Pre	AFQ-Y	1	219	-.226	.047	-.059	.000	.017	(-.092 | -.026)	.001**	2.22	.002	.174	(1.88 | 2.57)	< .001**
Post	AFQ-Y	1	219	-.085	.003	-.021	.000	.017	(-.053 | .012)	.218	1.55	-.002	.159	(1.24 | 1.86)	< .001**
Pre	CAMM	1	115	-.309	.088	-.141	.000	.038	(-.211 | .067)	.001**	5.48	-.002	.916	(3.66 | 7.26)	< .001**
Post	CAMM	1	115	-.013	-.009	-.006	.000	.040	(-.080 | .071)	.891	2.26	.001	.913	(.416 | 4.05)	.016*

Table S5b. Temporal Bias on Item Responses

				Residual		ANOVA		Durbin-Watson Test			Pre - Post Difference				
Condition	Test	df Hyp.	df Err.	*Mean*	*SD*	*F*	*p*	*D-W*	*bias*	S.E.	*Coeff.*	*t*	*df*	*d*	*p*
Pre	AFQ-Y	1	219	.000	1.26	11.8	.001**	2.56	-1.089	.126	B	1.58		.620	.127
Post	AFQ-Y	1	219	.000	1.22	1.58	.211	2.22	-.916	.129	c	2.84		1.11	.009**
Pre	CAMM	1	115	.000	1.26	12.2	.001**	1.60	-.513	.137	B	2.45		.999	.023*
Post	CAMM	1	115	.000	1.27	.019	.891	2.38	-.976	.167	c	2.49		1.01	.021*

AFQ-Y = Acceptance and Fusion Questionnaire for Youth; CAMM = Child and Adolescent Mindfulness Measure;

df = degree of freedom; Hyp. = hypotesis; Err. = errors; C.I. = confidence Interval; S.E. = Standard Error;

*R^2 = adjusted squared R. B = not normalized coefficient (item). c = constant; model: y= B*item+c; Coeff. = coefficients; d = effect size;*
All analysis conducted with Bootstrap N = 10.000 and BCa = Bootstrap Corrected Accelerated.
**p<.05; **p<.01 The difference is significant*

Table S6a. Within-Subjects Effects using Repeated Measures ANOVA

Test	Effect	*df Hyp.*	*df Err.*	*F*	*p*	η^2	Observed Power
AFQ-Y	Pre-Post	1.00	12.0	7.33	.019*	.379	.701
AFQ-Y	Items	6.38[c]	76.5 [c]	6.27	< .001**	.343	.999
AFQ-Y	Pre-Post x Items	75.1 [c]	2.18 [c]	.826	.558	.064	.314
CAMM	Pre-Post	1.00	11.0	1.03	.333	.085	.153
CAMM	Items	4.43 [c]	48.7 [c]	6.40	< .001**	.368	.989
CAMM	Pre-Post x Items	5.29	58.2	3.24	.011*	.227	.873

AFQ-Y = Acceptance and Fusion Questionnaire for Youth; CAMM = Child and Adolescent Mindfulness Measure;
df = degree of freedom; Hyp. = hypotesis; Err. = errors;
c = Greenhouse-Geisser Corrected for Non-Sphericity
**p<.05; **p<.01*

Table S6b. Within-Subjects Contrasts using Repeated Measures ANOVA

Test	Effect	Contrast	*df Hyp.*	*df Err.*	*F*	*p*	η^2	Observed Power
AFQ-Y	Pre-Post	Difference	1.00	12.0	7.33	.019*	.379	.701
AFQ-Y	Items	Linear	1.00	12.0	15.6	.002**	.565	.951
AFQ-Y	Pre-Post x Items	Linear	1.00	12.0	2.57	.135	.176	.314
CAMM	Pre-Post	Difference	1.00	11.0	1.03	.333	.085	.153
CAMM	Items	Linear	1.00	11.0	10.4	.008**	.486	.834
CAMM	Pre-Post x Items	Linear	1.00	11.0	7.25	.021*	.397	.689

AFQ-Y = Acceptance and Fusion Questionnaire for Youth; CAMM = Child and Adolescent Mindfulness Measure;
df = degree of freedom; Hyp. = hypotesis; Err. = errors;
c = Greenhouse-Geisser Corrected for Non-Sphericity
**p<.05; **p<.01*
